# Biventricular Outflow Obstruction Associated With Atrioventricular Septal Defects and Patent Ductus Arteriosus: An Extremely Rare Combination

**DOI:** 10.7759/cureus.12265

**Published:** 2020-12-25

**Authors:** Abdulrhman Alabdulgader, Abdullah A Alabdulgader, Metin Sungur, Tarek AlSayad

**Affiliations:** 1 Medicine, College of Medicine, King Faisal University, Al-Ahsa'a, SAU; 2 Congenital Heart Service, Prince Sultan Cardiac Center, Al-Ahsa'a, SAU; 3 Pediatric Medicine, Al-Azhar University, Cairo, EGY

**Keywords:** biventricular outflow obstruction, combined semilunar valves stenosis, atrioventricular septal defects, patent ductus arteriosus, balloon valvuloplasty, neonates, pediatric cardiology, congenital heart diseases (chds)

## Abstract

We present an extremely rare combination of biventricular outflow obstruction associated with atrioventricular septal defects and patent ductus arteriosus (PDA). Almost all the other published cases, including ours, were associated with other congenital cardiac lesions other than biventricular outflow obstruction. Most cases ended with poor outcomes. Our patient was a 55-day-old term female infant. She was managed by successful aortic balloon valvuloplasty with successful early outcome.

## Introduction

Congenital heart diseases (CHDs) are common in infancy, accounting for one third of all congenital disease [1]. In Saudi Arabia, CHDs account for 10.67 per 1,000 live births [2]. Ventricular septal defect (VSD) constitutes the most common symptomatic CHD [2]. However, from a prospective point of view, bicuspid aortic valve (BAV) is far more common, accounting for approximately 0.5%-2.0% of CHDs globally [3]. The isolated obstruction of semilunar valves is not uncommon, accounting for 12.4% of pulmonary stenosis and 2.5% of aortic stenosis to treat all CHDs in Saudi Arabia [4]. However, biventricular outflow obstruction rarely occurs. This diagnosis has been described in only 48 patients in the medical literature [4-16].

This article describes a case of a female infant patient who was diagnosed with biventricular outflow obstruction associated with VSD, atrial septal defects (ASD), and patent ductus arteriosus (PDA). To the best of our knowledge, this combination is unique in the medical literature. In this article, we are highlighting the importance of the accurate diagnosis of all described lesions, awareness of peculiar hemodynamic findings, and the importance of early intervention to avoid hemodynamic compromise. Failure to do so has the potential to lead to tragic outcomes [10].

## Case presentation

A 55-day-old term female infant with low birthweight (2 kg) was transferred to our tertiary cardiac center with a diagnosis of heart failure associated with a large VSD, moderate ASD, BAV, moderate PDA, and mild mitral regurgitation (MR). Upon arrival, the physical examination showed a nondysmorphic infant who was slightly tachypneic on nasal oxygen. Feeding was administered by a nasogastric tube. On cardiac examination, there was visible apical pulsation, no precordial bulge, and left parasternal thrill. On auscultation, the first and second heart sounds were normal. Grade 4/6 ejection systolic murmur was heard in the second and third left intercostal spaces, which were propagated all over the precordium.

**Figure 1:**
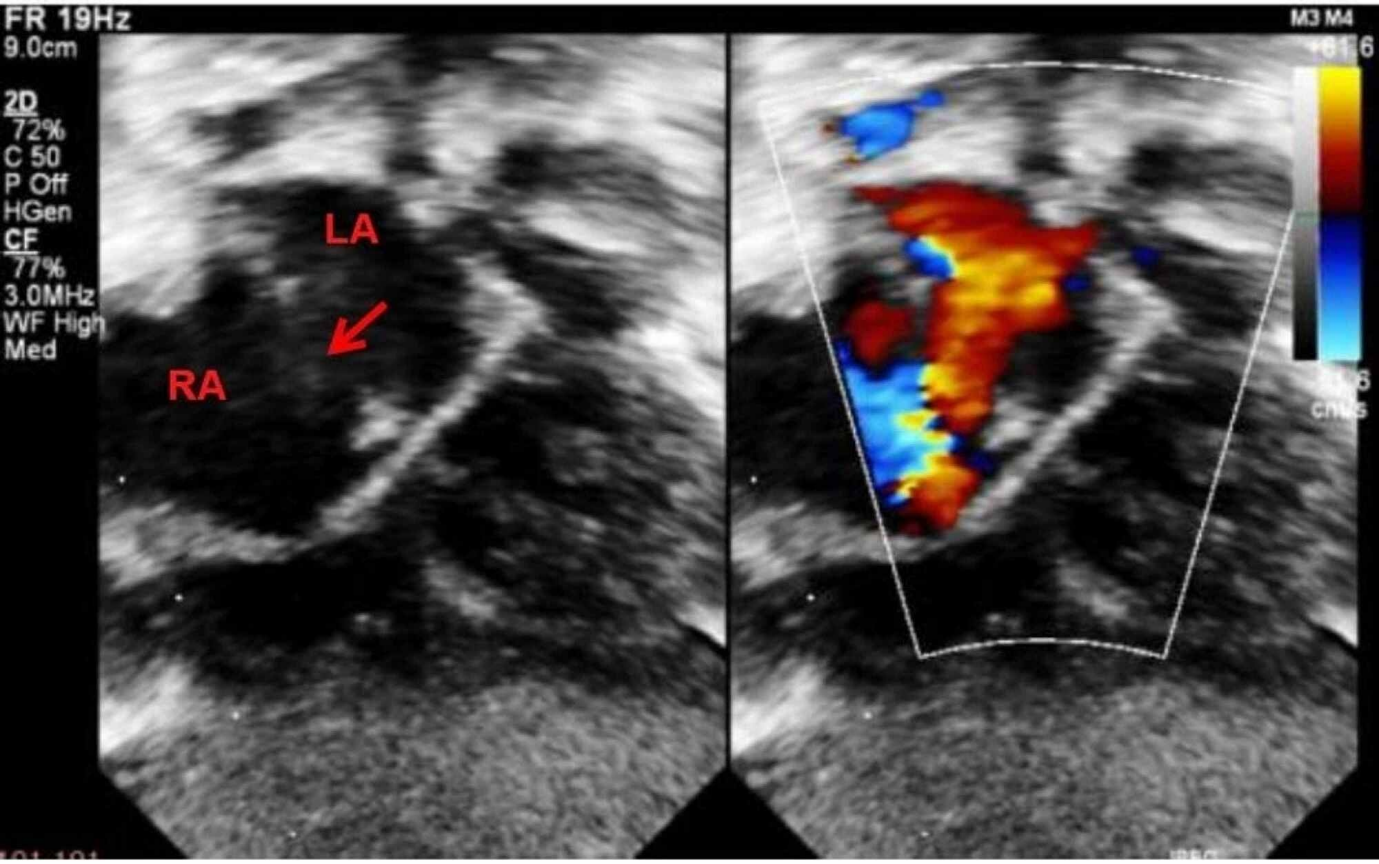
Two dimensional and color flow mapping of subcostal view showing atrial septal defect (arrow) with left to right shunt across. LA, left atrium; RA, right atrium

**Figure 2:**
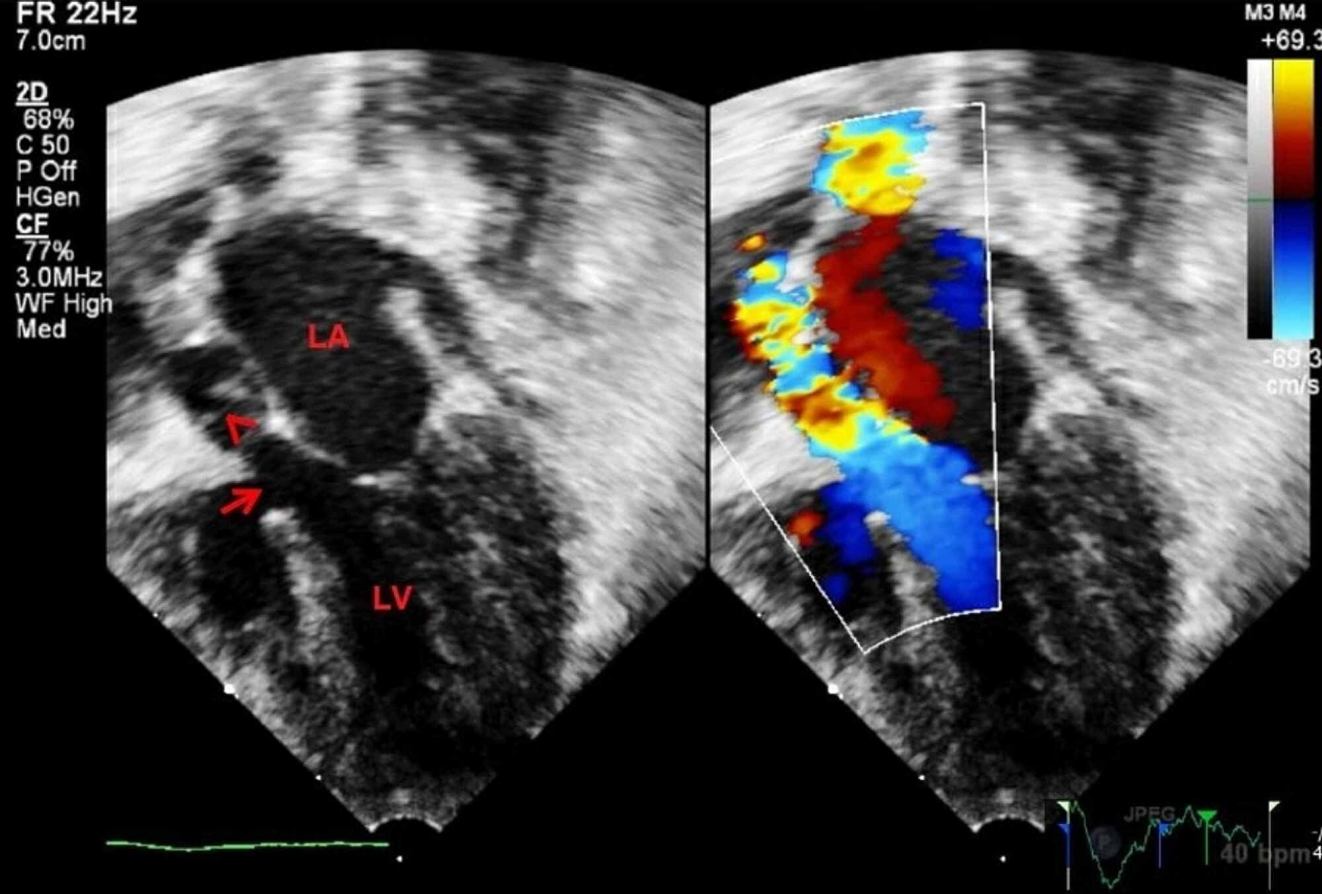
Two dimensional and color flow mapping of apical five chamber view showing outlet subaortic ventricular septal defect (arrow), smallish left ventricular outflow, and turbulent flow across stenotic aortic valve (arrow head). LA, left atrium; LV, left ventricle

**Figure 3:**
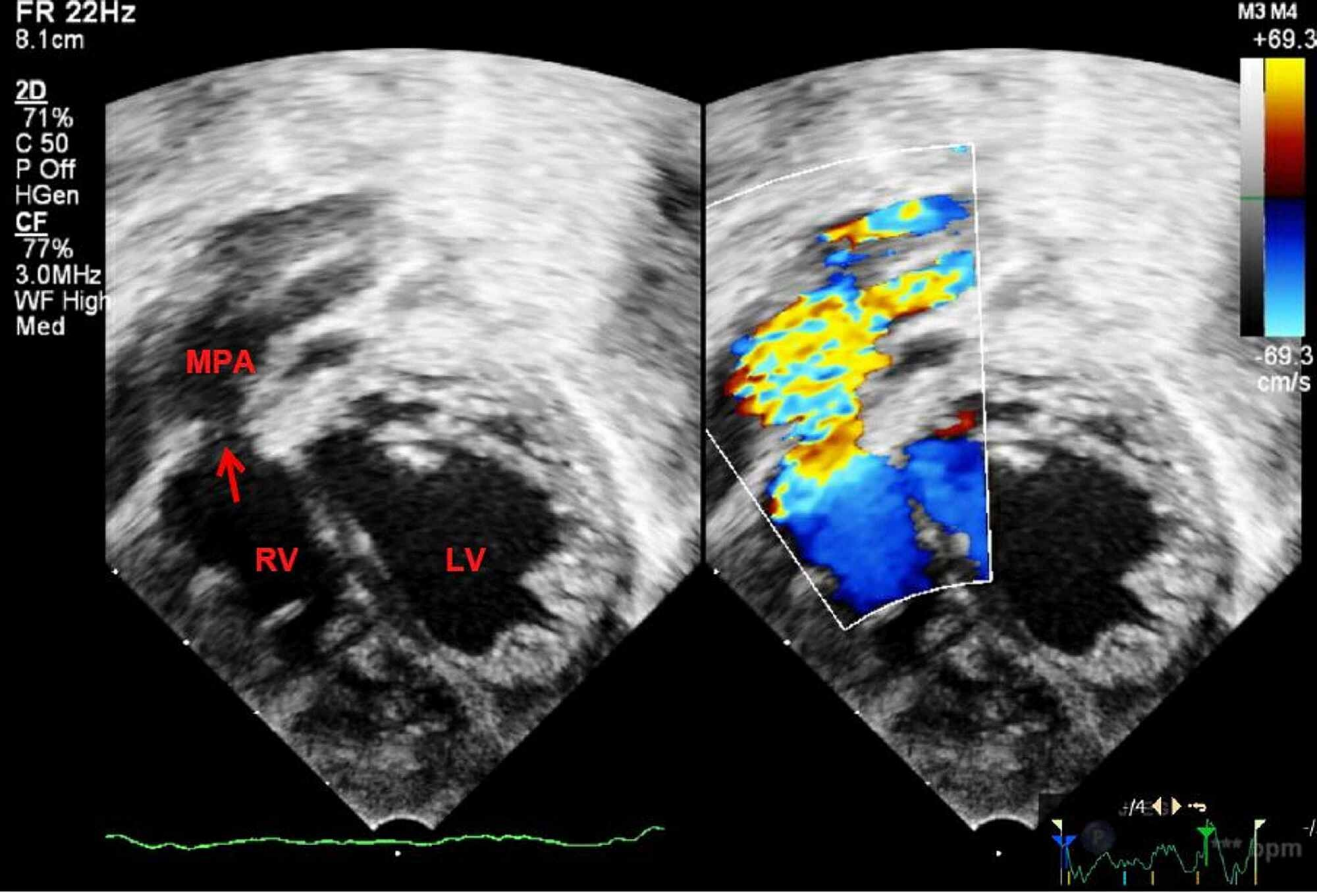
Two dimensional and color flow mapping of modified apical view showing turbulent flow across stenotic pulmonary valve (arrow). MPA, main pulmonary artery; LV, left ventricle; RV, right ventricle

**Figure 4:**
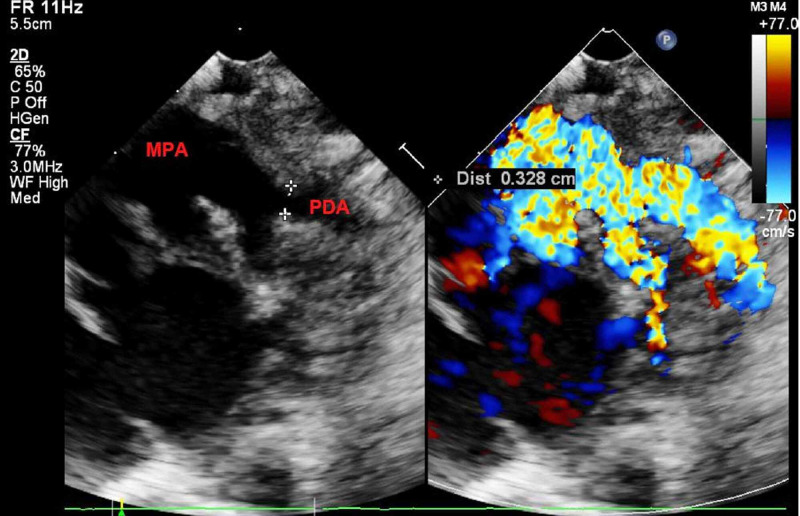
Two dimensional and color flow mapping of high parasternal “ductal” view showing large patent ductus arteriosus. MPA, main pulmonary artery; PDA, patent ductus arteriosus

The echocardiogram showed (Figures 1-4) a large secundum ASD (10 mm) with a left to right shunt, mild MR, moderate to severe tricuspid regurgitation, and dilated, hypertrophied left and right ventricles with good systolic function. Moderate (3.5 mm) VSD was observed at the subaortic outlet septum. The aortic valve was thickened and bicuspid with (6 mm annulus), and the peak pressure gradient (PG) across the aortic valve was 65 mmHg (a mean of 30 mmHg). A large PDA (3.6 mm) was observed at the pulmonary end with left to right nonrestrictive shunt. The pulmonary valve was thickened and dysplastic (8 mm annulus); a peak PG of 65 mmHg was noted. The patient was on furosemide, captopril, and spironolactone. 

 

**Figure 5:**
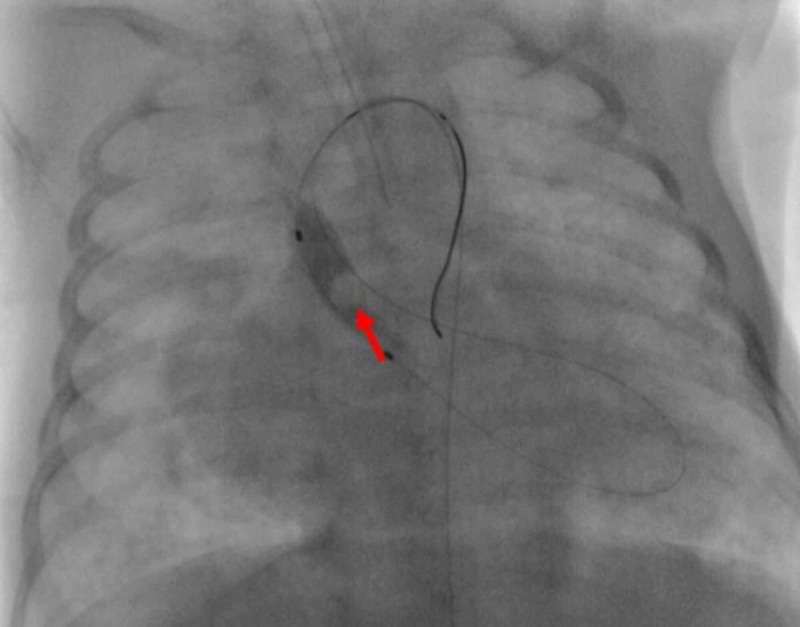
Antero-posterior fluoroscopic view during balloon valvuloplasty of the aortic valve with disappearance of the waist (arrow) followed by immediate pressure drop across the valve from 65 to 22 mmHg.

Left ventriculography was performed as part of cardiac catheterization and revealed a 4.2-mm aortic valve annulus. Balloon valvuloplasty of the aortic valve, a catheter-based intervention [Tyshak® Mini balloon (NuMED, Inc., New York), 4 mm × 2 cm, with three gradual inflation] was carried out (Figure 5). Successful aortic valvuloplasty was achieved when the PG dropped from 65 to 22 mmHg.

Postcardiac catheterization, she was extubated, hemodynamically stable and without heart failure, then transferred to the pediatrics ward. Two days later, she was transferred to the pediatric ICU and was re-intubated due to lung collapse. Two weeks later, the patient’s condition deteriorated. There was bleeding from the mouth and in the endotracheal tube suggesting acute lung injury. During this period of respiratory compromise, the cardiac parameters were normal. Despite aggressive management by adrenaline, cryoprecipitate, and factor 7 replacement, the patient developed severe bradycardia, desaturation, and hypotension. After 30 min of cardiopulmonary resuscitation, which failed to revive her, the patient was declared dead.

## Discussion

Biventricular outflow tract obstruction is rarely described in the medical literature; this diagnosis has been made in only 48 patients [4-16]. Richter described the first case in 1953 [4]. Most of the published cases presented with severe symptoms.

The age of presentation in the 48 cases varied, ranging from the prenatal period to 55 years. It was speculated that an early presentation indicated severe disease. Almost all the other published cases in the literature were associated with congenital cardiac lesions: ASD was present in nine cases [5-7, 10, 13-14], VSD was identified in eight cases [7, 9-11,15-16], and an atrioventricular canal defect was described in one case [12]. To the best of our knowledge, our case makes a unique combination to the medical literature on the topic in which the presence of combination of two left-to-right intracardiac shunts (ASD and VSD) and one left-to-right shunt at the great arteries level (i.e., PDA) were first described. This unique combination of biventricular outflow obstruction, associated with atrioventricular septal defects and PDA required special hemodynamic consideration. From an etiological point of view, such a combination provides a clue to understanding cardiac dysmorphogenesis processes in humans.

An evaluation was performed of the management of biventricular stenosis in the literature to identify the most optimal approach. Most cases published in the last century are characterized by poor outcomes, especially those prior to the era of catheter-based interventions. Specific management guidelines are not available in the medical literature due to the rarity of this condition. Clearly, evidence-based interventions rely on published case reports. Of the 48 cases, only nine of them were managed by balloon valvuloplasty. Ours was the 10th published case. In the current study, pulmonary valvuloplasty was not performed owing to the presence of a significant left-to-right shunt at three levels: ventricular, atrial, and great levels. The presence of native pulmonary valve stenosis is considered to be protective against pulmonary circulation overflow. Aortic valve PG dropped from 65 to 22 mmHg. This was accompanied by a significant improvement in the cardiac hemodynamic parameters and the patient’s general condition. The planned next step was to perform total repair: septal defects closure, PDA ligation, and pulmonary valvotomy. The patient's condition deteriorated because of sepsis with Gram-negative bacilli (Escherichia coli) despite the remarkable stable cardiac parameters. The current scenario is a strong indicator, for future cases to perform a meticulous search for associated extracardiac and systematic involvement. In our case, the deterioration in the infant’s condition is mainly attributed to sepsis, liver failure with increasing liver enzymes and deteriorated coagulation profile followed by pulmonary hemorrhage, and respiratory failure terminated by disseminated intravascular coagulation. Extracardiac involvement is significantly associated with CHD. This association is more pronounced with an increase in CHD complexity. Comparative studies carried out in our region on 2,020 cases, documented the involvement of other systems in one-third of CHD cases versus 10% in the controls. Anomalies of the central nervous system, hematological system, and gastrointestinal system were the most frequent associated extracardiac anomalies [17-18]. Meticulous search for associated extracardiac anomalies must be a critical part of the management plan in similar scenarios.

## Conclusions

Biventricular outflow obstruction is rarely described. Of the 48 published cases, only nine of them were managed by balloon valvuloplasty. Ours was the 10th published case. To the best of our knowledge, this combination of biventricular outflow obstruction ASD, VSD, and PDA were first described. Future cases should be recognized early with meticulous search for extracardiac anomalies to avoid tragic hemodynamic outcomes.
